# An Internet-Based Weight Loss Intervention Initiated by a Newspaper

**Published:** 2009-06-15

**Authors:** Lori Carter-Edwards, Lori A. Bastian, Mark Schultz, M. Ahinee Amamoo, Truls Østbye

**Affiliations:** Department of Community and Family Medicine, Duke University Medical Center; Duke University Medical Center and Veterans Administration Medical Center, Durham, North Carolina; Chapel Hill News, Chapel Hill, North Carolina. At the time of this research, Mr Schultz was affiliated with the *Herald-Sun*, Durham, North Carolina.; University of North Carolina School of Public Health, Chapel Hill, North Carolina; Duke University Medical Center, Durham, North Carolina

## Abstract

**Background:**

An estimated two-thirds of North Carolina residents are overweight or obese. Mass media, such as newspapers and the Internet, can be used to broadly convey health messages for weight loss.

**Context:**

Newspapers have traditionally been a primary source of health information for the general public. They may be uniquely suited to initiate and manage a community-based weight loss program by quickly reaching a broad readership.

**Methods:**

Participants in the 2005 Lose to Win weight loss challenge visited the *Herald-Sun* Web site and anonymously entered a nickname and identification number and reported their weight each week. Participants had access to weekly articles on diet and physical activity and 4 free educational seminars.

**Consequences:**

Of the 154 participants who self-reported weight at baseline and during the last week of the challenge, the mean weight lost was 5.9 lb.

**Interpretation:**

Results suggest that this challenge fostered health awareness and promoted weight loss in the community. Future interventions of this type should use strategies to increase participation and retention, improve the accuracy of reported weight, and evaluate long-term success of the program. This type of intervention may be a useful first step to reach residents who are interested in losing weight.

## Background

The high prevalence and health consequences of overweight and obesity in the United States are well documented (1), particularly in the Southeast. In North Carolina, approximately 40% of the population are overweight and an additional 25% are obese (2). Mass media has been used to help address this problem by accommodating people's busy lifestyles. The Internet is increasingly used to convey health messages for weight loss, whether targeted to people who are overweight or obese or to the general public (3-5). This medium can deliver lay health information quickly, cheaply, and 24 hours per day (6,7) and has the potential to provide the social support that is typically needed to maintain weight loss (4,5,8). In addition, it can empower healthy decision making (9) and offers anonymity that may encourage obese people, who may be embarrassed about their weight, to seek treatment.

## Context

Health information messages about weight loss may be disseminated through mass communication channels in various ways (10). Initially, the goal of these messages is to reach people who are ready to implement them (such as people who want to lose weight); ultimately, the goal is to improve the health of the population in general. Although limited information exists about the effectiveness of Web-based health interventions (11), the Internet may be an effective way to disseminate information about weight loss to large, diverse populations, including those with limited or inadequate access to health care.

Newspapers, traditionally a primary source of health information for the general public, are frequently accessed online (3). Online newspapers reach a wide audience quickly and easily. We report findings on an Internet-based weight loss program that was initiated by a newspaper, the *Herald-Sun*, in 2005 among residents of an urban North Carolina community.

## Methods

The *Herald-Sun* advertised its 15-week Lose to Win weight loss challenge, both in its print version and on its Web site; the *Herald Sun* is the most-read newspaper in the Durham, North Carolina, metropolitan statistical area (12). The challenge took place from January through May 2005. Adapted from the *Herald & Review* intervention in Decatur, Illinois, the *Herald-Sun* intervention sought to disseminate basic weight loss information and messages on lifestyle modification to a broad population, including people who may not receive these messages in a traditional health care setting. Participants visited the newspaper's Web site and anonymously entered a nickname (for 1 person or a group of people) during the first week of the challenge. The newspaper assigned each person or team an identification number and each team member a separate member identification number. For teams, leaders recorded each member's baseline weight at week 1, and thereafter, members logged on to the Lose to Win Web site and recorded their weight.

The *Herald-Sun* published a feature article each week in the "Healthy Living" section of the newspaper, accompanied by nutrition and physical activity articles from health experts and newspaper staff (Table). The Durham Fitness and Nutrition Council and fitness and exercise experts published weekly columns that appeared in both the print and online versions of the newspaper. The "Fitness Forum" provided general advice and practical information on topics related to diet and exercise. "Your Personal Trainer" and "For Bikers" (columns that were already part of the "Healthy Living" section) were published on alternating weeks during the challenge. The *Herald-Sun* published cumulative information on physical activity, including proper exercise form, so that participants and other readers could have the previous week's tips as a reference. The newspaper offered 4 free, informal, interactive educational seminars conducted at its main office by a team of 2 to 3 public health professionals.

To help kick off the challenge, the *Herald-Sun* solicited essays from potential participants about why they wanted to lose weight. The challenge's lead organizer and support staff reviewed approximately 40 essays for originality and level of enthusiasm. They selected and followed 5 participants, dubbed "role models," during the 15-week challenge; a summary of each role model's story was reported in the newspaper, and he or she received free, individualized nutritional counseling and personal fitness training from certified health care professionals. None of the other participants received incentives; the intent of the program was to reach readers who were independently motivated to adopt healthier lifestyles.

After Lose to Win ended in June 2005, we asked the lead organizer to meet with our research team because we wanted to understand more about the use of mass media in a weight loss challenge. We obtained the relevant articles published by the *Herald-Sun* and weight data that had been submitted to the Web site. The *Herald-Sun* granted us permission to clean and analyze the collected data and report the results. All data we received were deidentified, and this study did not require institutional review board approval.

A total of 705 people signed up for Lose to Win. We excluded from analyses 260 participants who did not have a baseline and at least 1 additional weight recorded during the challenge. We excluded another 291 who did not record weight in the final week of the challenge or who had duplicate identification numbers. Because participants were inconsistent about submitting weight throughout the challenge, we analyzed only those participants who had reported weights for both week 1 and week 15 (the final week), for a sample of 154 participants (or 21.8% of the original 705).

The raw database for the 2005 Lose to Win challenge was created and maintained throughout the intervention by staff at the *Herald-Sun*. It included the individual or team nickname, individual or team identification number, and weight entered for each week a person participated. No other data were collected. The number of participants who reported their weight varied from week to week. For each week, we calculated the mean weight of the participants who reported a valid weight that week (2 records with invalid weights — 11 lb and 12 lb — were excluded) (Figure). However, for comparative analysis, we computed mean change in weight from week 1 to week 15 for the 154 participants who made up the analysis sample. We also assessed whether the mean change in weight for participants in the analysis sample differed by whether they participated as a team or individually.

**Figure.**
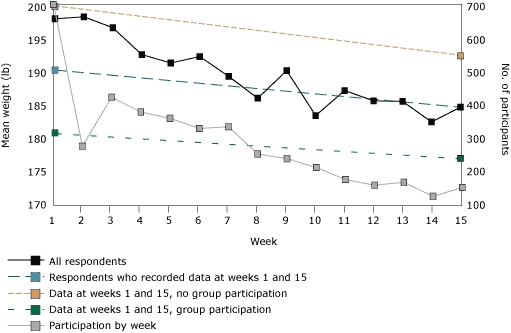
Mean reported weight of participants in the *Herald-Sun* 2005 Lose to Win challenge, Durham County, North Carolina. Mean weight is shown for all participants who reported weight each week. Lines are also shown for the subset of participants (n = 154) who reported weight on both the first and last week of the challenge, in total and by whether they participated with a group (n = 143) or independently (n = 11). Also shown is the week-by-week participation. A trend toward weight loss was reported among all subgroups, but standard deviations were wide and overlapped, so no between-group differences can be determined.

**Table:** 

**Respondents**	**Week of the Challenge**														
	**1**	**2**	**3**	**4**	**5**	**6**	**7**	**8**	**9**	**10**	**11**	**12**	**13**	**14**	**15**
All respondents	198.3	198.6	196.9	192.7	191.4	192.4	189.3	185.9	190.2	183.2	187.1	185.5	185.4	182.2	184.5
Respondents who recorded data at weeks 1 and 15	190.3														184.5
Data at weeks 1 and 15, no group participation	200.2														192.5
Data at weeks 1 and 15, group participation	180.5														176.5
Participation by week	705	277	423	379	360	330	335	254	240	214	178	161	170	128	154

## Consequences

Participants' mean weight steadily declined during the 15-week period of the challenge (Figure). However, the number of participants who submitted weight varied dramatically each week (range, 128-705). At week 1, the mean weight of the analysis sample was 190.3 lb (standard deviation [SD], 45.8 lb), and by week 15, participants had lost an average of 5.9 lb (SD, 7.5 lb). Although people who participated individually (n = 11) lost more weight on average (7.7 lb) than did those who participated as a group (n = 143, 5.7 lb), the difference did not reach significance (Wilcoxon 2-sample *t* test *P* = .23).

The 5 role models lost an average of 15.1 lb (SD, 6.2 lb). The role models also reported an overall reduction in dress or pant size and indicated that they enjoyed the program and found that the strategies helped them meet their needs. Some of the role models' comments were published in the *Herald-Sun* at the end of the challenge (13):

"My biggest challenge was not allowing my stressful and demanding life to derail my goal to live healthy, including regular exercise and eating a healthy and balanced diet…. If I want to grow and adapt to a positive lifestyle, I have to make it a priority."

"My biggest challenge was keeping the daily exercises as a top priority." She dealt with this by lining up other people as exercise buddies, such as a neighbor; 2 sessions a week with a Lose to Win trainer; and daily walks with people at work. She stuck with it, "because I wanted to see this change so badly, and it was something I was in complete control of."

Additionally, according to the lead organizer of the challenge, participants who attended the free seminars indicated that they appreciated the environment because it allowed them to openly ask questions and discuss and share personal issues related to weight and weight loss.

## Interpretation

Findings indicate that people who participated for the 15-week duration lost an average of nearly 6 lb, or approximately 3.2% of their initial weight. These results are similar to the 16-week results for the control group in a 52-week trial of a commercial Internet weight loss program (6). Participants were randomized to access either 1) online instructions on building a healthier diet, professionally moderated online meetings, online bulletin board support groups, a fitness instructor, e-mail reminders, and a 24-hour help desk (intervention group) (www.ediets.com, eDiets.com, Inc, Fort Lauderdale, Florida) or 2) a detailed, 16-step manual for modifying eating, activity, and thinking habits (control group) (14). Participants who received the manual lost more weight than did those in the Internet group (3.6% vs 0.9% of initial weight), which indicates that printed media may be more familiar to a general population and that the Internet is still an emerging medium for this type of education.

Internet-based weight loss interventions may require structured education to achieve maximum benefit (6,15). For the *Herald-Sun* Lose to Win challenge, specific details were provided on how to do a number of activities, including searching for the right foods and exercise instructions and tips. Additionally, attendance at interactive seminars supports the notion that some people may prefer onsite group sessions over Internet-based instruction (16).

Access to and use of a personal trainer, in conjunction with Internet-based instruction, may also help maximize program benefit. The 5 role models lost an average of 15 lb, which is comparable to the 18.3 lb lost in 6 months by participants in an online, therapist-led, structured weight loss intervention (16). These findings imply that support from others may be necessary to augment and maintain positive health behaviors.

As with other Internet- and non–Internet-based studies on weight loss (6), attrition was high. More than one-third of the original 705 participants did not submit their weight after week 1 of the challenge. Only 21.8% of the original sample submitted their weight at week 1 and week 15. However, this response rate is comparable to those of mailed surveys (17). The mean baseline weight of the 78.2% of participants who were not included in the analysis sample was 200.4 lb, more than 10 lb heavier than the mean baseline weight of the analysis sample. These people may have lost interest in participating, become frustrated with a perceived lack of progress over time, been less serious about losing weight, or never had any intention to implement behavior change. Regardless of their level of participation, the *Herald-Sun* exposed its readers to a series of health messages, which may have increased awareness of lifestyle modification strategies.

This challenge showed that an Internet-based program can promote weight loss among interested participants, even though participants have minimal or no direct contact with investigators. Other lessons learned are briefly described below.

### Increasing participation and retention

To increase participation, selected role models could promote the challenge. Role models could compete to see who could invite the most participants who submit complete data. To foster a comfortable, nonthreatening forum for participants to share ideas, successes, and milestones, future weight loss challenges may consider diversifying the health message and mode of dissemination by combining structured educational information with e-mail reminders, bulletin board support groups, and a help desk (15,18). To increase consistency in participants' reporting over time, weekly reminders could be available in print and on the Internet.

### Improving accuracy of reported weight

To increase the validity of self-reported weight, organizers should consider offering "weigh-ins" each week at popular locations in the community (eg, malls, pharmacies, supermarkets, recreation centers). To maximize anonymity, people could weigh themselves privately at weekly weigh-ins on a common scale provided by the newspaper, then submit their weight anonymously (identified only by participant number and team nickname). Although this would still be considered self-reported, it would increase accuracy without threatening participants' privacy. To further increase accuracy, participants could have their weight measured and submitted by a health professional.

### Evaluating the challenge's success over time

Evaluating success over time would require collecting and archiving additional information from participants. In addition to nicknames, identification numbers, and weight, participants could also report limited demographic information. Even if the program recorded only age, sex, and zip code of residence, this information would be useful in planning future weight loss interventions in the community. As a measure of the program's success, participants could be invited to report subjectively the usefulness of the information they received.

### Conclusions

The *Herald-Sun* 2005 Lose to Win weight loss challenge results provide information on the receptiveness of the public toward a program that was initiated by a newspaper and designed to use broad communication channels to disseminate information and conduct a weight loss intervention. Print and electronic newspapers are daily sources of information, and they may be effective in communicating to and accommodating the needs of a diverse population (19). That participants received no direct material incentives implies that they participated because they were motivated to change their behavior. Future weight loss studies in larger markets may provide further evidence that programs that disseminate weight loss information, in combination with step-by-step instructions, in print and on the Internet, may reach residents in populations that would otherwise be reluctant to seek traditional, professional care for overweight or obesity.

## Acknowledgments

We thank the participants of the 2005 Lose to Win challenge, the health professionals who contributed articles to the *Herald-Sun* or donated their time to conduct community education sessions, staff of the *Herald-Sun* for conducting the challenge and providing data, and Courtney Klemm at the *Herald & Review* for providing information about the Lose to Win challenge in Illinois. This work was supported by a grant from the National Institute of Diabetes and Digestive and Kidney Diseases (R01 DK64986). Dr Carter-Edwards was also funded in part by a National Institutes of Health Clinical Translational Science Award (CTSA UL1 RR024128-01).

## Figures and Tables

**Table.** Categories of Weekly Articles and General Topic Areas Covered in the *Herald-Sun* 2005 Lose to Win Challenge

**Category**	**Topic Areas**
Feature article	How to incorporate fitness into one's life Exercise Food behavior modification Empowerment and control Sustainability Steps to better health Food challenges Healthy alternatives
Durham Fitness and Nutrition Council article	Making excuses to exercise Cardiovascular fitness Using what you have in your home (no special equipment necessary to start exercising and tone muscle) The importance of using weights to tone muscle Measuring waist, hips, and bust to assess progress Selecting a trainer Exercise injury prevention
"Fitness Forum"	Exercising in a hotel Swimming Making gradual body changes to maintain weight loss Youth and weights Reducing bone loss through weight lifting Choosing appropriate workout shoes Using a dietitian *Trans* fats Using hills to work out Checking heart rate before exercising
"Your Personal Trainer" (biweekly, alternating with "For Bikers")	Athletic conditioning Chest exercises What you know about food (your nutrition IQ) American College of Sports Medicine standards for trainers Overexertion Exercising for seniors Effect of aerobics and stretching consistently Healthy fast food choices
"For Bikers" (biweekly, alternating with "Your Personal Trainer")	Falling off a bicycle is normal when learning to ride The effect of a railroad plan on biking The bus transit system's service for carrying bicycles on front of buses Unifying area bicycle commuters Safe traveling tips for bikers (including waiting for new bicycle trails to dry before using them) Where to test new bicycles
Exercises (step-by-step descriptions, including photographs)	Squat series (basic and chair) Push up series (wall, full form, and knee) Lunges Chest presses Military shoulder presses Pelvic presses Bent-over fly exercises Hip and thigh series (standing inner and outer thigh workouts) Biceps curls Lateral raises Triceps kickbacks and stretches Lower leg series (heel and toe raises) Abdominals Seated gluteus stretches Spinal stretch series (spinal twist, cat, and side stretches)
